# Variability in gut mucosal secretory IgA in mice along a working day

**DOI:** 10.1186/s13104-018-3213-0

**Published:** 2018-02-05

**Authors:** Patricia Burns, Sofia Oddi, Liliana Forzani, Eduardo Tabacman, Jorge Reinheimer, Gabriel Vinderola

**Affiliations:** 1 Instituto de Lactología Industrial (INLAIN, UNL-CONICET), Facultad de Ingeniería Química, Universidad Nacional del Litoral, Santiago del Estero 2829, 3000 Santa Fe, Argentina; 2 Departamento de Matemática, Facultad de Ingeniería Química, Universidad Nacional del Litoral, Santiago del Estero 2829, 3000 Santa Fe, Argentina; 3 Somalogic, Inc, 2945 Wilderness Pl, Boulder, CO 80301 USA

**Keywords:** IgA, Intestine, Circadian rhythm, Mice, Variability

## Abstract

**Objective:**

To assess the variability of secretory immunoglobulin A (S-IgA) in the lumen and feces of mice along a working day.

**Results:**

Mice were maintained under a 12 h light–dark cycle, light period starting at 8 AM. S-IgA was determined in feces and intestinal content (after one or three washes) at three points along the day: at the beginning, in the middle and at the end of the light period (ELP). Significant reduction in the content of S-IgA in the small intestine fluid and in feces was observed at the end of the light cycle, which coincides with the end of a regular working day (8 PM) in any given animal facility. It was also observed that three washes of the small intestine were more effective than one flush to recover a significant higher amount of S-IgA, with the smallest coefficient of variation observed by the ELP. A smaller CV would imply a reduced number of animals needed to achieve the same meaningful results. The results may be useful when designing animal trials for the selection of probiotic candidates based on their capacity of activating S-IgA, since it would imply a more rational use of experimental animals.

## Introduction

Probiotics are live microorganisms that when administered in adequate amounts confer a health benefit on the host [1]. One criterion when selecting probiotics is their capacity to stimulate secretion of secretory IgA (S-IgA) [2, 3], the immunoglobulin in charge of exclusion of pathogens [4]. Different probiotic strains have shown the capacity of enhancing mucosal IgA in mice [5–7]. This capacity has been linked to the protection against gut pathogens [8, 9]. In those assays mice are fed the strain for different feeding periods (2, 5, 7 or 3, 6, 10 days) and animals are sacrificed on the same day. This implies sampling many animals along the same working day. Previous results (not published) showed unexpected dispersion of S-IgA values in mice of the same group, impairing the observation of differences between groups. Immune parameters oscillate rhythmically in the day [10]. In humans, salivary IgA reflect circadian rhythmicity, which peak during sleep [11]. In rats, fecal IgA exhibited a clear diurnal rhythm [12]. We aimed to assess the variability of S-IgA in the lumen and feces of mice along a working day.

## Main text

### Materials and methods

#### Animals

Twenty-four 6-week old male BALB/c mice (20 ± 1 g) were used (CMC-ICiVet-Litoral, CONICET–UNL). Animals were kept for 7 days before the trial at 21 ± 2 °C, 55 ± 2% humidity and 12 h light–dark cycle. The light period started at 8 AM. The trial was approved by the Ethical Committee for Animal Experimentation (FCV-UNL), protocol 291/16, June 26th 2016.

#### Sampling of feces and intestinal content

Animals were sampled (8 animals/group in a period of 15 min) at three time-points: at the beginning, in the middle and at the end of the light period (named BLP, MLP, ELP). Before sacrifice, feces were collected, weighed, diluted 100× (1% (v/v) anti-protease cocktail (P8340, Sigma) in PBS, homogenized (Ultra Turrax T8, Ika Labortechnik, Staufen, Germany), centrifuged (5000×*g*, 10 min, 4 °C) and the supernatant was frozen at − 70 °C for S-IgA quantification by ELISA [13], using Sigma reagents (M-8769 anti-mouse IgA, Fast OPD P-9187, M1421 IgA and A 4789, anti-mouse IgA—peroxidase antibody.

Animals were anesthetized intraperitoneally (ketamine, xylazine and acepromazine) and sacrificed by cervical dislocation. Small intestine was removed and flushed with 5 mL of PBS supplemented with 1% (v/v) anti-protease cocktail. The intestinal content was vortexed and sampled for S-IgA quantification. The remaining intestinal content suspension was used to flush the small intestine twice. Intestinal fluid suspensions were centrifuged (2000×*g*, 30 min, 4 °C) and frozen at 70 °C for S-IgA [13].

#### Statistical analysis

R© software (2.12.2 version) was used (R Development Core Team, 2011). The coefficients of variation (CV) of the logarithm of the values of S-IgA were compared for each combination of sampling point/number of flushes against the smallest CV observed in the intestinal fluid or in feces. ANOVA was applied to data and the differences between means were detected by Tukey post hoc test. Data were considered significantly different when p < 0.05.

## Results and discussion

### Need for data transformation

Many of the parametric tests and models commonly used (linear models, t test, ANOVA) are based on a normal distribution of data. One way to deal with this is to transform data, usually by a log transformation, as antibody titres do not follow normal distribution [14]. The Box-Cox test helps determine the best transformation procedure out of a family of power transforms (which includes the logarithm for the power parameter of 0). We applied this test to study S-IgA. This gives a 95% confidence interval for the power parameter of (− 1/2, 1/2), therefore suggesting that a log transformation is suitable. For ease of interpretation we use log in base 10. The statistical justification for log transformation is to use the proper methodology, as analyzing data in a transformed scale can change the significance of a test. Let us assume for a moment that the small intestine was flushed once and we want to compare if there is a difference between the BLP and ELP groups. The right methodology, under normality of data, would be to apply a t test, then the p value obtained is 0.01173, whereas if a log-transformation of data is applied, then p value is 0.006914.

### Analysis of the variability of data

For detecting differences in the content of S-IgA among groups, it is necessary to get rid of the so-called spurious variability. S-IgA may be highly variable among individuals of the same group [15], reducing the effectiveness of sample size, and thus creating the necessity of more samples to achieve a meaningful statistical power. It is then useful to study the intrinsic variability of the data to find out means to reduce it. In this study, the time of the day when mice were sacrificed and the number of flushes of the small intestine were suspected to produce variability in the measurements of S-IgA.

The coefficient of variation (CV) is a dimensionless parameter defined as the ratio of the standard deviation to the mean, and is considered a useful indicator of relative consistency of data. The sample size and power of many common tests of differences are related to the measurements and the variability through the CV. The larger the CV, the smaller the power. In this work, the smallest CV in the intestinal lumen was observed for the group ELP-TW, whereas in feces it was observed for the group BLP (Table 1).

**Table 1:** Coefficients of variation of log S-IgA in the intestinal lumen after one or three washes and in feces

Sampling point	CV (log S-IgA)		
	One wash (OW)	Three washes (TW)	Feces
BLP	0.1048	0.0797	0.050
MLP	0.0496	0.0490	0.088
ELP	0.0588	0.0326	0.101

It was then determined which CVs were statistically bigger than the smallest ones in each type of sample. A permutation test was used [16, 17]. There are two versions of this test (BII and BIII) but since the minimum CV is less than 0.6, version BII is the recommended one. Except for the first wash made in the MLP and at the ELP, all CVs were statistically bigger (p < 0.05) compared to corresponding smallest CV. In feces, the CVs in the sampling points MLP and ELP were not statistically different compared to the smallest value observed at the BLP, but this can be due to a lower power of the test.

In case of a simple t test of difference of means, the sample size needed to detect a fixed percentage change of ratio of means depends quadratically on the coefficient of variation [18]. For example, in the intestinal fluid, the CV of the BLP-OW group (0.1048) was 3.2 times that of the ELP-TW group (0.0326). Therefore, the sample size needed in a t test of difference of means should be 10 times larger to find differences within the same level of confidence if mice are sampled at the BLP and making one flush of the small intestine, compared to the fact of collecting intestinal fluid at the BLP and flushing the small intestine three times. A smaller CV would imply a smaller group of animals, contributing then to the 3R’s principle (Replacement, Reduction and Refinement).

In relation to the dispersion of individual values of S-IgA in the intestinal fluid, a maximum of less than four folds of difference were observed in this work, whereas in the work of Grewal et al. [15] this factor reached up to more than 30 folds. In feces, the animal that contained more S-IgA in the BLP group (the less dispersed one), presented a value 2.7 times higher than the mouse that presented the smallest value, being this factor around 4.6 for the other two groups. This is in line with a previous work [15], where a difference of 4.8 folds was observed.

### S-IgA in the intestinal fluid and in feces along a working day

For S-IgA in intestine, if the data were technical and completely independent (that is one value obtained from each mouse), an ANOVA procedure with two factors (sampling point and number of flushes) could be used. However, the same mouse was used for sampling both flushes, then it was used a mixed-effects linear model that takes into account the dependency of the response on the flush.

**Fig. 1:**
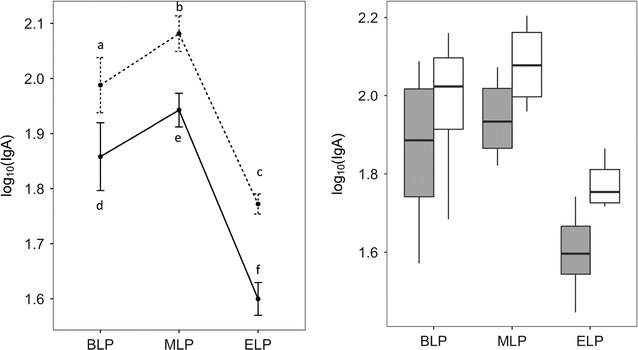
Means ± standard deviation (left graph) and boxplot (right graph) of log of S-IgA (µg/mL) in the small intestine content of mice (8 animals/group) sacrificed at the beginning (BLP), in the middle (MLP) or at the end (ELP) of the light period. Luminal S-IgA was recovered by flushing the small intestine once (black line on left graph and grey boxes on the right graph) or three times (dashed line on left graph and white boxes on the right graph). Points indicated with* a*,* b* and* c* are significantly different (p = 2 × 10^−16^) from points indicated with* d*,* e* and* f*, respectively. Point* c* is significantly different from points a and* b* and point* f* is significantly different from points* d* and* e* (p = 1 × 10^−4^). ANOVA was applied to the data and the differences between means were detected by Tukey’s post hoc test. Data were considered significantly different when p < 0.05

There was no interaction between sampling points and the number of flushes (p = 0.4439), which means that one or three flushes resulted in the same trend of results along the day (Fig. 1, line plot). The ANOVA coefficients of both factors were significant (p = 2.9 × 10^−12^ for the sampling point and 1.7 × 10^−5^ for the number of flushes). A post hoc Tukey test for comparison of means showed that three flushes resulted in a statistically higher (p = 2 × 10^−16^) amount of IgA than one flush, for all sampling points. Additionally, the amount of S-IgA present in the intestinal lumen at the ELP was significantly lower than in the MLP or at the BLP (p = 1 × 10^−4^), but the groups MLP and BLP were not statistically different (p = 0.253) (Fig. 1, box plot).

**Fig. 2:**
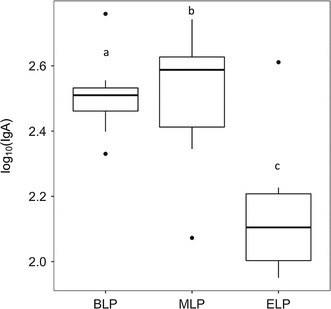
Boxplots for log (S-IgA) (µg/g) in feces of mice sacrificed at the beginning (BLP), in the middle (MLP) or at the end (ELP) of the light period. Point c is significantly different from points* a* (p = 0.003) and* b* (p = 0.003), while points* a* and* b* are not significantly different (p = 1.000). ANOVA was applied to data and the differences between means were detected by Tukey’s post hoc test. Data were considered significantly different when p < 0.05

The content of S-IgA in feces (Fig. 2) depended on the sampling point (p = 0.001208). The post hoc Tukey test showed no difference between BLP and MLP groups (p = 0.9997430), whereas S-IgA was significantly lower at the ELP compared to the BLP (p = 0.0032681) or to the MLP (p = 0.0031073).

Mice are nocturnal animals and the cyclicity of hormones and several immune parameters correlates with the pattern of the animal locomotor activity-resting. The immune parameter that peaks at one time of day for a diurnal species peaks about 12 h later for a nocturnal one [19]. In humans, S-IgA in saliva peaks during sleep [11], whereas in this work, S-IgA peaked by the middle of the light period, where animals are quieter than during the dark period. The mouse model of activation of S-IgA for *Salmonella* infection prevention has been largely used for assessing the probiotic potential of strains [20, 21]. Mice infected with *S*. *Typhimurium* were colonized to higher levels and developed a higher proinflammatory response during the early rest period for mice, showing that a functional clock is required for optimal *S*. Typhimurium colonization [22]. The results shown here may help choosing the proper moment along the day for challenging mice when using the salmonellosis murine model [23].

It is interesting to note that the smallest CV was observed after three flushes at the ELP, whereas in feces the smallest CV was observed early in the morning (BLP). Considering that the gastrointestinal transit time in mice, from oral gavage, is close to 8 h [3], then it is likely that the IgA present in the lumen by the ELP is also present in feces at the BLP, coinciding that they both displayed the lowest CVs. In order to reduce data dispersion and make experiments more powerful, fecal samples may be harvested at the BLP, whereas sacrifices may take place at its end. In this sense, fecal IgA could be a suitable parameter to monitor the activation of the gut immune response, as S-IgA in feces correlates with those in the lavage samples [15]. An ethical reduction in the number of animals used could be achieved, as sacrifice would proceed as soon as a peak in S-IgA is observed in feces, instead of using several groups of mice for different feeding periods.

The influence of the circadian rhythm on S-IgA was observed in this work. A significant reduction in the content of S-IgA in the intestinal fluid and in feces was noticed at the end of the light cycle, which coincides with the end of a regular working day (8 PM) in any given animal facility. Three washes of the small intestine were more effective than one flush to recover S-IgA, with the smallest CV observed by the ELP. A smaller CV implies less animals to get the same meaningful results. These results may be useful when designing animal trails for the selection of probiotics based on their capacity of activating S-IgA, since it would imply a more rational use of experimental animals contributing to the 3R’s principles.

## Limitations

We were not able to measure IgA during the dark period. The profile of certain cytokines (IL-6, IL10, IL-2, IL-12, IFNγ and TNFα) in the small and large intestine are parameters of interest that will be assessed in future full research works.

## Abbreviations

S-IgA: secretory immunoglobulin A
BLP: beginning of the light period
MLP: middle of the light period
ELP: end of the light period
OW: one wash
TW: three washes
CV: coefficient of variation

## References

[1] Hill C, Guarner F, Reid G, Gibson GR, Merenstein DJ, Pot B (2014). Expert consensus document. The International Scientific Association for Probiotics and Prebiotics consensus statement on the scope and appropriate use of the term probiotic. Nat Rev Gastroenterol Hepatol..

[2] Galdeano CM, de Moreno de LeBlan A, Carmuega E, Weill R, Perdigón G (2009). Mechanisms involved in the immunostimulation by probiotic fermented milk. J Dairy Res.

[3] Padmanabhan P, Grosse J, Asad ABMA, Radda GK, Golay X (2013). Gastrointestinal transit measurements in mice with 99mTc-DTPA-labeled activated charcoal using NanoSPECT-CT. EJNMMI Res..

[4] Brandtzaeg P, Bjerke K, Kett K, Kvale D, Rognum TO, Scott H (1987). Production and secretion of immunoglobulins in the gastrointestinal tract. Ann Allergy.

[5] Generoso SV, Viana M, Santos R, Martins FS, Machado JAN, Arantes RME (2010). *Saccharomyces cerevisiae* strain UFMG 905 protects against bacterial translocation, preserves gut barrier integrity and stimulates the immune system in a murine intestinal obstruction model. Arch Microbiol.

[6] Frece J, Kos B, Beganović J, Vuković S, Šušković J (2005). In vivo testing of functional properties of three selected probiotic strains. World J Microbiol Biotechnol.

[7] Ya T, Zhang Q, Chu F, Merritt J, Bilige M, Sun T (2008). Immunological evaluation of *Lactobacillus casei* Zhang: a newly isolated strain from koumiss in Inner Mongolia, China. BMC Immunol..

[8] Vinderola G, Matar C, Perdigón G (2007). Milk fermented by Lactobacillus helveticus R389 and its non-bacterial fraction confer enhanced protection against Salmonella enteritidis serovar Typhimurium infection in mice. Immunobiology.

[9] Benyacoub J, Pérez PF, Rochat F, Saudan KY, Reuteler G, Antille N (2005). Enterococcus faecium SF68 enhances the immune response to *Giardia intestinalis* in mice. J Nutr.

[10] Man K, Loudon A, Chawla A (2016). Immunity around the clock. Science.

[11] Wada M, Orihara K, Kamagata M, Hama K, Sasaki H, Haraguchi A (2017). Circadian clock-dependent increase in salivary IgA secretion modulated by sympathetic receptor activation in mice. Sci Rep..

[12] Eriksson E, Royo F, Lyberg K, Carlsson H-E, Hau J (2004). Effect of metabolic cage housing on immunoglobulin A and corticosterone excretion in faeces and urine of young male rats. Exp Physiol.

[13] Rodrigues AC, Cara DC, Fretez SH, Cunha FQ, Vieira EC, Nicoli JR (2000). Saccharomyces boulardii stimulates sIgA production and the phagocytic system of gnotobiotic mice. J Appl Microbiol.

[14] Manikandan S (2010). Data transformation. J Pharmacol Pharmacother..

[15] Grewal HM, Karlsen TH, Vetvik H, Ahrén C, Gjessing HK, Sommerfelt H (2000). Measurement of specific IgA in faecal extracts and intestinal lavage fluid for monitoring of mucosal immune responses. J Immunol Methods.

[16] Amiri S, Zwanzig S (2010). An improvement of the nonparametric bootstrap test for the comparison of the coefficient of variations. Commun Stat Simul Comput..

[17] Cabras S, Mostallino G, Racugno W (2006). A nonparametric bootstrap test for the equality of coefficients of variation. Commun Stat Simul Comput..

[18] van Belle G, Martin DC (1993). Sample size as a function of coefficient of variation and ratio of means. Am Stat.

[19] Płytycz B, Seljelid R (1997). Rhythms of immunity. Arch Immunol Ther Exp.

[20] Castillo NA, de Moreno de LeBlanc A, Galdeano CM, Perdigón G (2012). Probiotics: an alternative strategy for combating salmonellosis. Food Res Int.

[21] Zacarías MF, Reinheimer J, Forzani L, Grangette C, Vinderola G (2014). Mortality and translocation assay to study the protective capacity of *Bifidobacterium lactis* INL1 against *Salmonella Typhimurium* infection in mice. Benef Microbes..

[22] Bellet MM, Deriu E, Liu JZ, Grimaldi B, Blaschitz C, Zeller M (2013). Circadian clock regulates the host response to Salmonella. Proc Natl Acad Sci.

[23] Curtis AM, Bellet MM, Sassone-Corsi P, O’Neill LAJ (2014). Circadian clock proteins and immunity. Immunity.

